# Short‐term changes in and preoperative factors affecting vaulting after posterior chamber phakic Implantable Collamer Lens implantation

**DOI:** 10.1186/s12886-021-01963-x

**Published:** 2021-05-06

**Authors:** Qiu-Jian Zhu, Wen-Jing Chen, Wei-Jian Zhu, Hai-Xiang Xiao, Man-Hui Zhu, Lie Ma, You Yuan, E. Song

**Affiliations:** Department of Ophthalmology, Lixiang Eye Hospital of Soochow University, Jiangsu 215021 Suzhou, China

**Keywords:** ICL implantation, Vault, Sulcus to sulcus, Crystalline lens thickness, Prediction

## Abstract

**Background:**

To describe the very early vault changes in the first month after Implantable Collamer Lens (ICL) implantation and to evaluate the effect of preoperative biometric factors on vault.

**Methods:**

Eighty-three eyes from eighty-three subjects with complete data who met follow-up requirements were recruited in this retrospective study between May 2019 and March 2020. We quantitatively assessed the postoperative vault at 2 h, 1 day, 1 week, and 1 month following implantation. Associations between the postoperative vault and age, ICL size, spherical equivalent (SE), axial length (AL), central corneal thickness (CCT), flat keratometry (K), steep K, mean K, anterior chamber depth (ACD), crystalline lens thickness (LT), white-to-white (WTW) diameter obtained by three devices, horizontal and vertical sulcus-to-sulcus (STS) diameter, bright and dark pupil sizes (BPS and DPS) and DPS-BPS were investigated using Spearman’s correlation analysis and stepwise multiple regression analysis.

**Results:**

The mean vault values at 2 h, 1 day, 1 week, and 1 month after ICL implantation were 672.05 ± 30.72, 389.15 ± 28.33, 517.23 ± 30.76 and 530.12 ± 30.22 μm, respectively. Significant differences were found in the vault values at 2 h, 1 day and 1 week after the operation. The ICL size (β = 0.942; *p* < 0.001), followed by horizontal STS (β = -0.517; *p* < 0.001), crystalline LT (β = -0.376; *p* < 0.001) and vertical STS (β = -0.257; *p* = 0.017), significantly influenced the vault at 1 month after the operation. The multiple regression equation was expressed as follows: central vault (µm) = -1369.05 + 657.121 × ICL size- 287.408 × horizontal STS − 432.497 × crystalline LT − 137.33 × vertical STS (adjusted R^2^ = 0.643).

**Conclusions:**

After ICL implantation, the vault decreased and then increased, but it did not return to the vault value 2 h after surgery. The ICL size, horizontal and vertical STS and crystalline LT are key factors for predicting postoperative vaulting.

**Supplementary Information:**

The online version contains supplementary material available at 10.1186/s12886-021-01963-x.

## Background

The Implantable Collamer Lens (ICL; STAAR Surgical Co, Monrovia, California) is a safe and effective option to correct myopia [1–4]. With no corneal excision and few high-order aberrations, it is often the first choice for surgical correction of high myopia [5, 6]. Although ICL implantation offers outstanding benefits, postoperative complications have been reported, most of which were associated with the vault (distance between the posterior ICL surface and anterior crystalline lens surface) inappropriateness [7, 8]. A low vault may lead to mechanical contact with the lens or inadequate aqueous humour circulation, accounting for the high incidence of anterior capsular opacity and cataract formation [9, 10]. Conversely, a high vault can cause excessive mechanical contact between the ICL and iris, leading to inflammation and increased intraocular pressure [11, 12]. Additionally, the occurrence of pigment dispersion syndrome, iris atrophy, and acute angle-closure glaucoma has been associated with a high vault [13–15].

Many factors can influence the vault after ICL implantation. Lee et al. [16] found that horizontal compression of the ciliary sulcus is a key factor in vault formation, but it could not effectively predict vault. Trancon et al. [17] and Zeng et al. [18] believed that the anatomy of the crystalline lens could affect vaulting after surgery. Additionally, many studies have found that changes in pupil size are closely associated with changes in vaulting [19–21]. Unfortunately, insufficient studies exist to integrate these factors to predict postoperative vaulting.

Very early vaulting changes, which are often used in contralateral eye surgery strategies, are often ignored by researchers. Therefore, the present study revealed the early change process in vaulting through observations from 2 h to 1 month after ICL implantation and established preoperative biometric factors that might contribute to vault formation and prediction.

## Methods

### Study design and participants

This retrospective study was approved by the Lixiang Eye Hospital of Soochow University Institutional Review Board and adhered to the tenets of the Declaration of Helsinki. All the patients were examined, treated and followed at the refractive surgery centre of Lixiang Eye Hospital between May 2019 and March 2020. Eighty-three eyes from eighty-three subjects with complete data were recruited for this study. Informed consent was obtained from each subject before surgery.

The inclusion criteria for this study included patients aged 18–45 years, patients with myopia between − 0.50 and − 21.00 DS, patients with astigmatism between 0 and − 6.00 DC, patients with an anterior chamber depth (ACD, the distance between the corneal endothelium and anterior surface of the lens) equal to or greater than 2.80 mm, and patients with an endothelial cell density greater than 2000 cells/mm^2^. None of the patients had ciliary body cysts, obvious cataracts, glaucoma or retinal disease history, or systemic diseases. One eye was randomly selected for the subjects who had surgery on both eyes.

### Preoperative examination

All the patients had undergone complete ophthalmic examinations, including uncorrected and best corrected distance visual acuity evaluation, slit-lamp microscopy, tonometry (noncontact tonometer; NT-530, Nidek Co., Ltd., Aichi, Japan), and fundus examination using a three-mirror lens. The refractive dioptre was measured using a standard phoropter and converted into the spherical equivalent (SE), which was calculated as the original spherical dioptre plus a half of astigmatism. Flat K, steep K, mean K, central corneal thickness (CCT) and ACD were obtained using a Scheimpflug camera (Pentacam, Oculus, Germany). The bright and dark pupil sizes (BPS and DPS) were measured using an OPD-Scan III device (Nidek Technologies, Gamagori, Japan). Axial length (AL) and crystalline lens thickness (LT) were obtained using an IOLMaster 700 (Carl Zeiss Meditec AG, Jena, Germany). Ultrasound biomicroscopy (UBM; SW-3200 L; SUOER, Tianjin, China) equipped with a 50-MHz transducer was performed to measure the horizontal and vertical sulcus-to-sulcus (STS) diameter after instillation of proparacaine (Alcaine; Alcon, Fort Worth, TX, USA). Horizontal corneal diameter and white-to-white distance (WTW) measurements were performed using a Pentacam, OPD-Scan III device and IOLMaster 700.

All examinations were performed in a room with constant temperature and humidity controlled by an air conditioning system. Phoropter, Pentacam and OPD-Scan III examinations were conducted in the darkroom, and all other examinations were conducted under standard room lighting conditions. Each test was performed by the same experienced physician or technician.

### Surgical procedure

ICL implantation followed a standard procedure and was performed by the same experienced surgeon (You Yuan, corresponding author). After topical anaesthesia (proxymetacaine hydrochloride; Baisite, Ruinian phar., Nanjing, China) was applied and hyaluronic acid (Qisheng, Qisheng phar., Shanghai, China) was injected into the anterior chamber via a 3.0-mm temporal corneal incision using an injector cartridge, an ICL V4c model (VICMO or VTICMO) was implanted and then placed in the posterior chamber. Next, the hyaluronic acid was completely removed from the eye using a manual Irrigation/Aspiration (I/A) instrument. All surgeries were uneventful, and no intraoperative complications were observed. Following surgery, tobramycin 0.3 % dexamethasone 0.1 % eye drops (Tobradex; Alcon, USA) were administered four times daily for the first 5 days, three times daily for the next 5 days and two for the last 5 days. The power calculations for the ICL were performed according to the manufacturer’s guidelines using a modified vertex formula [22]. All ICLs were placed at 10° horizontally (0° to 10° or 170° to 180°, regardless of the right or left eye), and only four size changes (12.1, 12.6, 13.2, and 13.7 mm) were available for use.

### Follow‐up

All subjects had undergone vault measurement using a Pentacam at 2 h, 1 day, 1 week and 1 month after ICL implantation. The patient placed their chin on the chin rest and their forehead against the forehead strap and was asked to open both eyes and fixate on the blue fixation target in the centre of the black background. Fifteen Scheimpflug image enhancement models were used to obtain anterior segment images. The image quality was checked using the quality factor value for each eye. Two experienced technicians blinded to the treatment groups independently measured the centre vault value in the Pentacam Scheimpflug image using the device’s built-in image analyser. The vault measurement was centred on the optical axis and appeared as a white dashed line on the screen. Each technician obtained three measurements, which were averaged. If the difference between the two technicians was less than 30 μm, the average value of the six measurements was included in the analysis, and if the difference value was equal to or more than 30 μm, the measurements were repeated until the difference was less than 30 μm.

### Statistical analysis

SPSS 18.0 (IBM Corp., New York, NY, USA) was used to perform the data analysis and the Kolmogorov-Smirnov test was performed for all measurement data. The data with a normal distribution were expressed as means ± standard deviation (SD); otherwise, the data were expressed as medians and quartiles. Repeated measures analysis of variance was used to calculate the vault change, and post hoc comparisons among time points were performed using Bonferroni correction. A paired sample t test was used to assess the correlation between the postoperative vault at 2 h and 1 day and postoperative vault at 1 month. Spearman’s correlation analysis and stepwise multiple regression analysis were used to examine associations between 1-month ICL vaulting and the other variables. The independent variables included age, ICL size, SE, AL, CCT, flat K, steep K, mean K, ACD, LT, WTW obtained using three devices, horizontal and vertical STS, BPS, DPS, and DPS-BPS. A p-value of less than 0.05 was considered significant.

## Results

The average age of the subjects was 27.21 ± 5.07 (range: 18 to 38) years, and 32.53 % (27/83) of the subjects were male. Table 1 summarizes the baseline clinical characteristics of the 83 eyes and descriptive data for preoperative variables.

**Table 1:** Baseline clinical characteristics of the study eyes (83 eyes)

Characteristics	Mean ± SD	Range
Age, years	27.21 ± 5.07	18 to 38
Sex (male/female)	27/56	
Laterality (right/left)	43/40	
Refractive errors (D)		
Spherical	-7.48 ± 3.11	-1.25 to -15.75
Cylindrical	-1.80 ± 0.94	0 to -5.0
Spherical equivalent	-8.36 ± 3.18	-2 to -17
Keratometric value (D)		
Flat K	42.72 ± 1.59	38.5 to 46.5
Steep K	44.59 ± 1.93	39.9 to 49.2
Mean K	43.69 ± 1.72	39.5 to 47.8
STS diameter (mm)		
Vertical	11.93 ± 0.52	10.64 to 13.30
Horizontal	11.51 ± 0.49	10.25 to 12.96
IOP (mm Hg)	13.98 ± 3.06	7.30 to 21.00
AL (mm)	26.83 ± 1.30	23.93 to 29.73
ACD (mm)	3.38 ± 0.27	2.80 to 3.76
WTW diameter (mm)		
Pentacam	11.65 ± 0.38	10.8 to 12.9
OPD-Scan III	11.86 ± 0.44	10.77 to 13.26
IOLMaster 700	12.01 ± 0.38	11.2 to 13.3
Pupil size (mm)		
Bright	3.53 ± 0.59	2.39 to 5.11
Dark	6.37 ± 1.06	3.94 to 8.56
Dark-bright	2.84 ± 0.77	0.90 to 4.60
ICL size (12.1/12.6/13.2/13.7)	7/38/35/3	
Crystalline LT (mm)	3.70 ± 0.24	3.11 to 4.22
CCT (mm)	525.22 ± 32.62	458 to 589

*STS *sulcus-to-sulcus, *WTW *white-to-white, *IOP *intraocular pressure, *ACD *anterior chamber depth

**Fig. 1:**
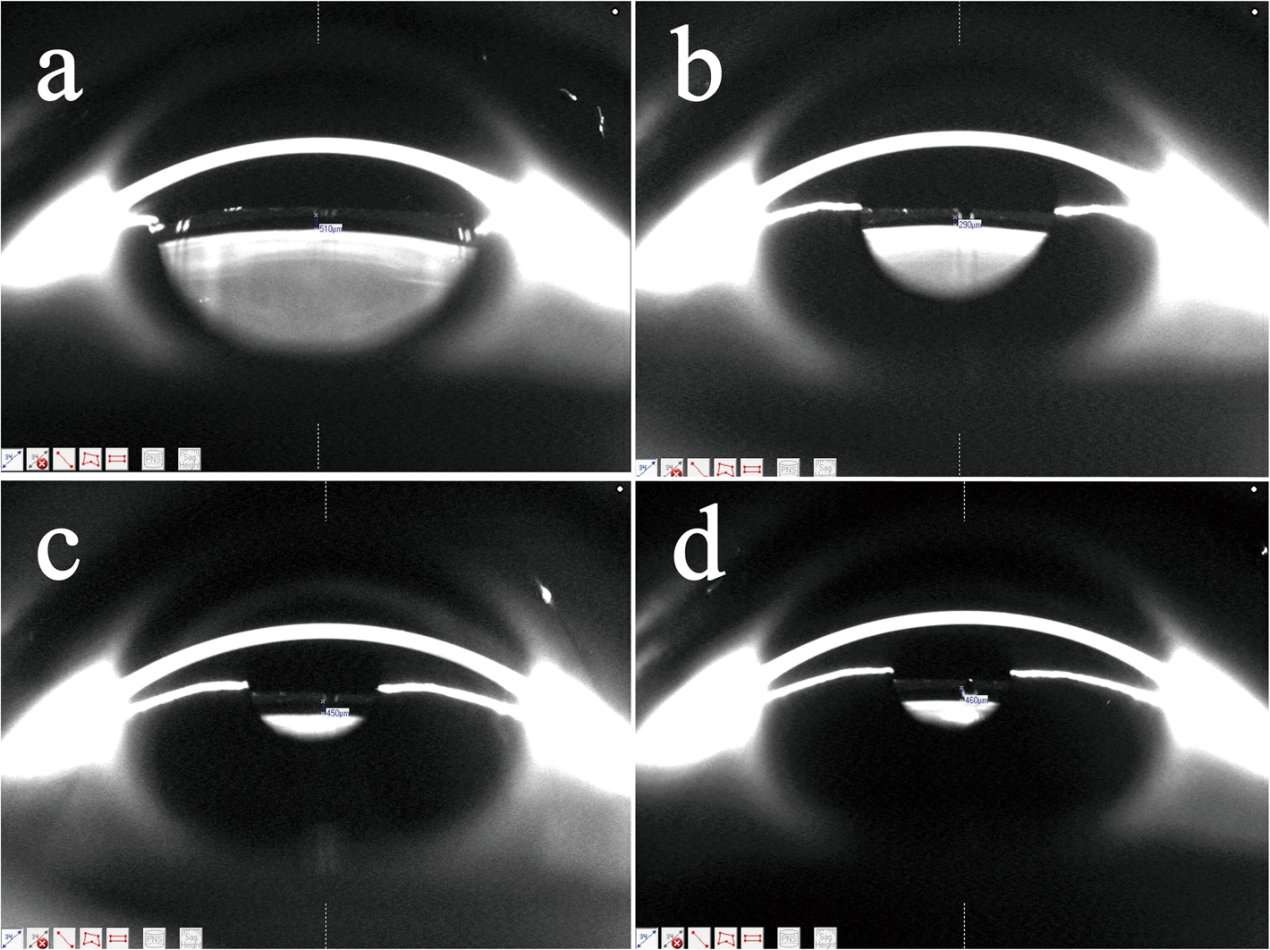
Vault changes within 1 month after ICL implantation. **a**, **b**, **c**, and **d** represent 2 h, 1 day, 1 week, and 1 month after surgery. The vault values were 510, 290, 450 and 460 μm, respectively

**Fig. 2:**
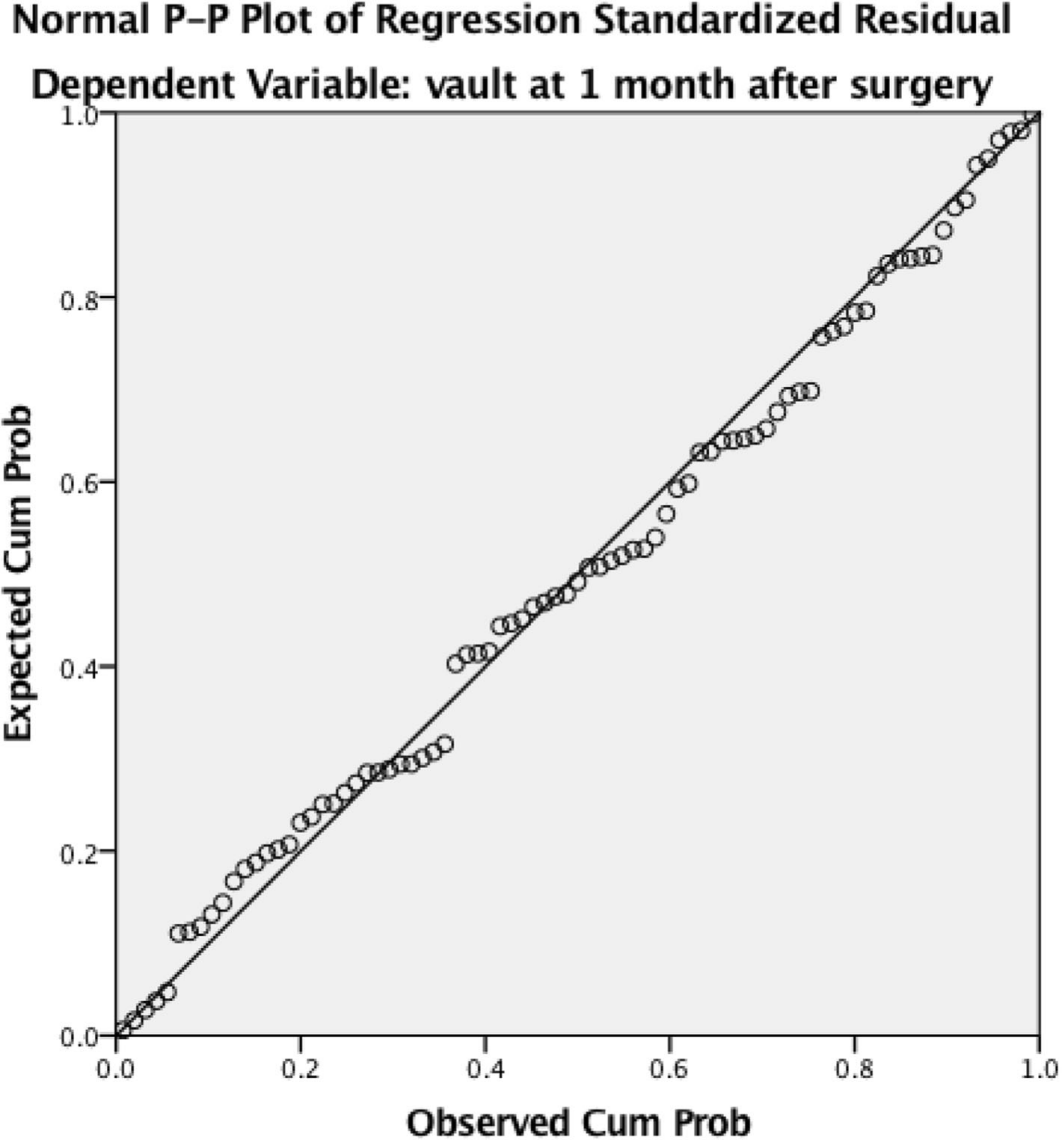
Normal P-P plot of the regression standardized residual, which shows excellent prediction accuracy

Table 2 shows the repeated measures analysis of the variance results of the vault at each time point after ICL implantation. The vault value was 672.05 ± 30.72 μm at 2 h after surgery, decreased to 389.15 ± 28.33 μm at 1 day then increased to 517.23 ± 30.76 μm at 1 week after surgery. No significant difference was found in the vault between 1 week and 1 month after surgery. Figures 1 and 2 show vault changes within one month after ICL implantation.

**Table 2:** Repeated measures analysis of variance of the vault at each time point after ICL implantation

	2 h	1 day	1 week	1 month	Correlation with time (*P* values)^a^
Vault (µm)	672.05 ± 30.72	389.15 ± 28.33	517.23 ± 30.76	530.12 ± 30.22	< 0.001
*P* value^b^	Ref.	< 0.001	< 0.001	< 0.001	
		Ref.	< 0.001	< 0.001	
			Ref.	0.448	

*Ref.* reference mean value

^a^ANOVA with repeated measures, significant *P* values of the repeated factor ‘time’

^b^Significant *P* values of the comparisons between the mean values with respect to the reference time, using the post hoc Bonferroni test

The paired sample correlation between vault values at 2 h and 1 month after surgery was 0.879 (*p* < 0.001) and that between vault values at 1 day and 1 month after surgery was 0.823 (*p* < 0.001). The paired difference between vault values at 2 h and 1 month after surgery was 141.93 ± 136.66 μm (95 % confidence interval [CI]: 112.09 μm, 171.77 μm; *p* < 0.001) and that between 1 day and 1 month after surgery was − 140.96 ± 159.36 μm (95 % CI: -175.76 μm, -106.17 μm; *p* < 0.001).

According to Spearman’s correlation analysis, the vault at 1 month after surgery was positively correlated with ACD, WTW and the ICL size and negatively correlated with crystalline LT (Table 3; all *p* < 0.05). Table 3 also shows the results of stepwise multivariate regression analysis. The explanatory variables relevant to vaulting were crystalline LT (standardized partial regression coefficient [β] = -0.376; *p* < 0.001), the ICL size (β = 0.942; *p* < 0.001), horizontal STS (β = -0.517; *p* < 0.001) and vertical STS (β = -0.257; *p* = 0.017). The multiple regression equation was expressed as follows: central vault (µm) = -1369.05 + 657.121 × ICL size − 287.408 × horizontal STS − 432.497 × crystalline LT − 137.33 × vertical STS. The formula requires further validation. The R, R^2^ and adjusted R^2^ of the model were 0.814, 0.660 and 0.643, respectively. Figure 2 shows that the prediction formula has excellent prediction accuracy.

**Table 3:** Spearman’s correlation analysis and stepwise multiple regression analysis between the 1-month ICL vault and other variables

	Spearman’s correlation		Multivariable analysis (constant=-1369.05; R = 0.813; R^2^ = 0.660; adjusted R^2^ = 0.643)		
Variables	r	*P* value	Partial regression coefficient (B)	Standardized partial regression coefficient (β)	*P* value
Age	-0.313	0.004			
SE	0.019	0.861			
Keratometric value					
Flat K	0.147	0.185			
Steep K	0.070	0.531			
Mean K	0.133	0.232			
STS					
Vertical	-0.140	0.206	-137.330	-0.257	0.017
Horizontal	0.060	0.592	-287.408	-0.517	< 0.001
IOP	-0.157	0.156			
AL	0.001	0.990			
ACD	0.261	0.017			
WTW diameter					
Pentacam	0.288	0.008			
OPD-Scan III	0.242	0.028			
IOLMaster 700	0.255	0.020			
Pupil size					
Bright	0.103	0.353			
Dark	0.140	0.207			
Dark-bright	0.129	0.246			
ICL size	0.450	< 0.001	657.121	0.942	< 0.001
Crystalline lens thickness	-0.603	< 0.001	-432.497	-0.376	< 0.001
Central corneal thickness	-0.201	0.068			

*STS *sulcus-to-sulcus, *WTW *white-to-white, *IOP *intraocular pressure, *ACD *anterior chamber depth

## Discussion

More than 120,000 ICL implantation surgeries are performed in over 60 countries annually, making the vault, an indicator of postoperative safety, particularly important [23]. In the current study, we aimed to assess the early changes in the ICL vault in the first month, starting at 2 h after ICL implantation. We analysed preoperative variables, including patient age, the ICL size, SE, AL, CCT, flat K, steep K, mean K, ACD, crystalline LT, WTW obtained by three devices, horizontal and vertical STS, BPS and DPS and their difference, to identify factors that influenced or may be used to predict the postoperative ICL vault.

Our results demonstrated a significant decrease in the vault postoperatively from 2 h to 1 day after implantation, followed by an increase from 1 day to 1 week; at 1 month after surgery, the vault was still lower than that 2 h after surgery. Because most surgeons perform ICL implantation at different times, an early vault after surgery is the reference for the ICL size selection for the contralateral eye. However, few studies have described changes in the vault within 24 h after surgery [21]. According to the paired sample t test, a vault at both 2 h and 1 day after surgery showed a good correlation with a vault at 1 month after surgery. However, a vault at 1 month after surgery was lower than that at 2 h and higher than that at 1 day after surgery. We speculated that residual viscoelastic agent played a critical role in the relatively high vault value at 2 h after surgery because the vault then decreased after removal of the viscoelastic agent by aqueous humour circulation. Garcia-Feijoo et al. [24] demonstrated that ICL haptics were usually ultimately located in the ciliary sulcus or ciliary body, while Choi et al. [25] demonstrated that 64.7 % of phakic IOL haptics were fixated in the ciliary sulcus. However, by analysing the full-scale UBM of 134 eyes, Zhang et al. [26] found that the ICL haptics in most cases were not in the ciliary sulcus and that different haptic positions had a significant influence on postoperative vaulting. For example, the eyes with haptics on the top of the ciliary sulcus were likely to have a high vault value, while those with one haptic on the ciliary process and another haptic in the ciliary body had a low vault value. We speculated that the change in the position of the haptics may also explain the change in the vault value after surgery. Additionally, previous studies have shown that changes in pupil size were associated with postoperative vaulting [27–30]. Lee et al. [19] believed that pupil constriction creates anteroposterior vectors through iris constriction, which exerts pressure on the ICL. Because the V4C ICL has a central hole, pressure equilibrium is quickly achieved between the front and rear surfaces of the ICL, facilitating this process (the fountain effect of “aquaport”). Thus, the effect of the iris has the net effect of pushing ICL into the lens, followed by a reduction in the central vault. Recently, Kato et al. [20] and Gonzalez-Lopez et al. [31] demonstrated that the ICL vault can be significantly decreased by light-induced pupil constriction. Thus, we speculated that pupil constriction due to the disappearance of the effect of the mydriatic agent played a very important role in vault reduction within 1 day after surgery. Finally, several studies have proven that the morphology of the crystalline lens also affects the vault after ICL implantation [32–34]. The ICL vault is affected by changes in the crystalline lens rise (CLR) caused by accommodation or light condition changes [20, 35, 36]. We hypothesized that the morphological changes in the crystalline lens caused by accommodation after surgery might also be a reason for a decreased vault. Regarding the change from 1 day to 1 month after surgery, our results were highly consistent with those by Chen and colleagues, who believed that changes in the pupil size and position of haptics were the main reasons for the results [21].

 According to our results, the vault value at 1 month after surgery was positively correlated with ACD, WTW and ICL size and negatively correlated with crystalline LT. However, we believe that such results are of little clinical significance because the ICL size was an important factor affecting postoperative vaulting. In our study, ICL size was not a continuous variable, so it had a significant impact on the results of Spearman’s correlation analysis. We are confident in the results of multivariate analysis. We found that the ICL size, followed by horizontal STS, crystalline LT and vertical STS, significantly influenced 1-month postoperative vaulting. Previous studies have shown that the ciliary sulcus is vertically oval [37–39]. Because the ICL has a flat plate design and a certain width, the supporting points of the lens are located between the horizontal and vertical ciliary sulcus but closer to the horizontal position. Therefore, the influence of the horizontal STS distance on postoperative vaulting is greater than that of the vertical STS (standardized partial regression coefficient: -0.517 vs. -0.257). As described previously, the morphology of the crystalline lens has a certain influence on vaulting after ICL implantation. Most recent studies have focused on the effect of the CLR on vaulting after surgery [31, 34–36, 40]. However, the measurement of the CLR is relatively complex. Qi et al. [41] demonstrated that the crystalline LT had an important influence on the postoperative vault, a finding that is highly consistent with our results, and crystalline LT can be easily obtained using an IOLMaster 700. A correlation exists between the CLR and crystalline LT, which must be further verified in subsequent studies. Our regression formula used the crystalline LT as one of the independent variables with a very high degree of fitting (the R, R^2^ and adjusted R^2^ of the model were 0.814, 0.660 and 0.643, respectively), indicating that the crystalline LT is an excellent predictive variable. Our results also showed that pupil size can influence the postoperative vault, but the vault cannot be predicted by the preoperative pupil size, including BPS, DPS or the difference between the two.

Conventionally, the manufacturer’s recommendation for ICL size refers to only two parameters: WTW and ACD (Visian ICL Product Information: Visian ICL For Myopia. Available at http://www.accessdata.fda.gov/cdrh_docs/pdf3/p030016c.pdf)). According to our results, neither WTW measured by any instrument nor ACD was a reliable predictor of postoperative vaulting, which was also the consensus of many similar studies [34, 35, 42]. Lee et al. [19] obtained the following regression formula after multivariate linear regression analysis of 236 patients with 12.6-mm crystal implantation: central vault (µm) = − 0.784 + (0.171 × preoperative ACD) + (0.038 × preoperative pupil size) + (0.017 × preoperative AL). Unfortunately, the fitting degree of this formula was very low (R^2^ = 0.144), only one size of ICL was included in this study, and the axial direction of ICL placement was not considered, which would affect the results. Chen et al. [21] developed the following regression formula in their study: ICL V4 central vault (µm) = (386.51 × ACD) − 718.77, ICL V4c central vault (µm) = (503.43 × ACD) − 1075.64. Similarly, the low fitting degree (adjusted R^2^ = 0.320 and 0.297) and small sample size (38 eyes for the V4 group and 39 eyes for the V4c group) make the results unsatisfactory. Recently, Igarashi et al. [42] developed a relatively good prediction formula based on the angle-to-angle (ATA) measurement: postoperative vault (mm) = 660.9 × (ICL size [mm] – ATA [mm]) + 86.6. However, the fitting degree of the adjusted R^2^ (0.41) was still not completely satisfactory. The NK formula developed by Nakamura et al. [34, 43] is likely the most accurate prediction formula thus far. The formula considers the distance between scleral spurs (Anterior Chamber Width, ACW) and the CLR as independent variables; the R^2^ of the multiple regression was 0.68, and the adjusted R^2^ was 0.666. In subsequent validation, a moderate vault was achieved in 92.1 % of cases by applying the formula [43]. The regression formula in this study has some similarities with the NK formula. For example, they have similar fitting degrees (adjusted R^2^ of 0.666 vs. 0.643). The CLR and crystalline LT both describe the morphology of the lens, and the distance between scleral spurs and the horizontal and vertical STS describe the anatomical morphology of the posterior chamber. Additionally, the sample size of our study was larger (83 eyes vs. 46 eyes) than theirs, and the crystalline LT was easier to measure. To identify the presence of ciliary body cysts, UBM is an important preoperative examination before ICL implantation [18, 44]. Therefore, our formula may be applied conveniently, without an additional anterior segment Optical Coherence Tomography (AS-OCT) examination. However, further validation is needed before clinical use.

There are certain limitations in this study. First, although the sample size was larger than those in some similar studies, it still must be supplemented in subsequent studies. Second, we only developed the prediction formula, and verification of the formula must be performed. We intend to publish later. Our study was conducted only among Han Chinese, and further study is needed to determine whether ethnic differences will impact the results. Finally, vault measurement may itself be variable because it is performed manually, and we did not measure the agreement of the interobservers. We used the method of averaging two independent measurers after the measured difference was less than 30 μm.

## Conclusions

In summary, we described very early changes in the ICL vault in the first month, starting at 2 h after ICL implantation, and we found that the ICL size, followed by the horizontal STS, crystalline LT and vertical STS, significantly influenced the vault 1 month after surgery. We hope our findings and new formulas will be helpful for surgeons when choosing the appropriate ICL size.

## Supplementary Information

**Additional file 1.**

## Data Availability

We have already uploaded the data in the current study as supplementary material. If someone wishes to request the data from this study, please contact You Yuan (Corresponding author).

## Abbreviations

ICL: Implantable Collamer Lens
STS: Sulcus to sulcus
WTW: White to white
IOP: Intraocular pressure
ACD: Anterior chamber depth
BPS: Bright pupil size
DPS: Dark pupil size
